# Polyrotaxanes as emerging biomaterials for tissue engineering applications: a brief review

**DOI:** 10.1186/s41232-020-00136-5

**Published:** 2020-11-11

**Authors:** Arun Kumar Rajendan, Yoshinori Arisaka, Nobuhiko Yui, Sachiko Iseki

**Affiliations:** 1 Section of Molecular Craniofacial Embryology, Graduate School of Medical and Dental Sciences, Tokyo Medical and Dental University, 1-5-45 Yushima, Bunkyo-ku, Tokyo, 113-8549 Japan; 2 Department of Organic Biomaterials, Institute of Biomaterials and Bioengineering, Tokyo Medical and Dental University, 2-3-10 Kanda-Surugadai, Chiyoda, Tokyo, 101-0062 Japan

## Abstract

The field of tissue engineering and regeneration constantly explores the possibility of utilizing various biomaterials’ properties to achieve effective and uneventful tissue repairs. Polyrotaxanes (PRXs) are supramolecular assemblies, which possess interesting mechanical property at a molecular scale termed as molecular mobility. This molecular mobility could be utilized to stimulate various cellular mechanosignaling elements, thereby altering the cellular functions. Apart from this, the versatile nature of PRXs such as the ability to form complex with growth factors and peptides, numerous sites for chemical modifications, and processability into different forms makes them interesting candidates for applications towards tissue engineering. This literature briefly reviews the concepts of PRXs and molecular mobility, the versatile nature of PRXs, and its emerging utility towards certain tissue engineering applications.

## Background

The search and designing of new biomaterials that can impart unique bio-interface functions and cellular modulations are never ending [1, 2]. It has been shown that various mechanical properties of the biomaterial interfaces can be utilized to modulate different cellular responses [3–5]. Some of the biomaterial features that could initiate cellular mechanosignaling include the elasticity of the materials, stiffness, hardness, and topographical features such as groves, micro/nanopillars, geometry, and shape of cellular adhesion surfaces [6–9]. Furthermore, it has also been shown that the application of forces, either in static or cyclic form, in certain directions can bring about various changes in the cellular response and functions [10, 11]. These mechanosignaling factors have been extensively studied and utilized in various biomaterial scaffolds for the purpose of tissue regeneration. One of the recent approaches is exploring the relative movement of molecules to one another, termed as molecular mobility, for modulating various cellular functions [12–14]. Molecular mobility is termed as the movement of certain molecules in relation to other molecules such as hinged flexing movement, sliding or rotation of ring-shaped molecule along an axle molecule, and many more [15]. Polyrotaxanes (PRXs) are one of the molecular assemblies which can exhibit molecular mobility.

## Polyrotaxanes and molecular mobility

**Fig. 1:**
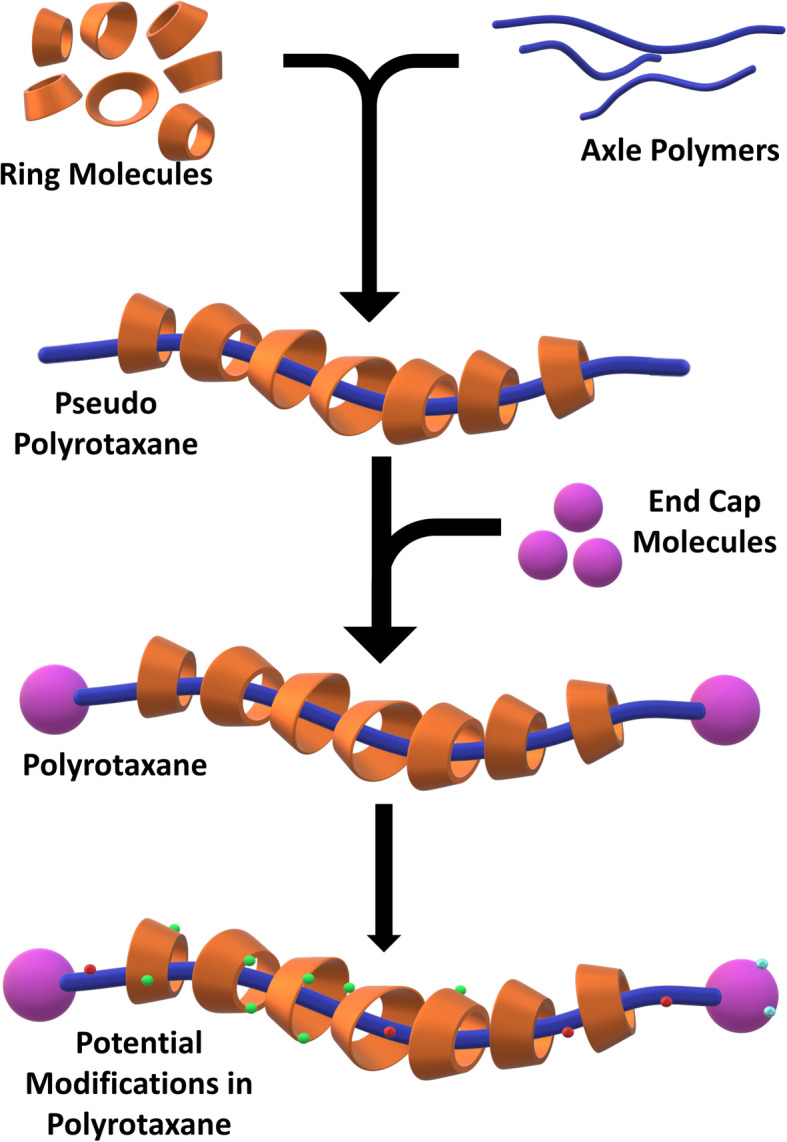
One of the schematic of synthesis of the PRX supramolecular assembly. The long axle polymers (blue) are threaded into the ring-shaped molecules (orange) to form the pseudo PRX molecules. Then, end cap molecules (purple) are added to prevent the dethreading of ring molecules, thereby resulting in PRXs. This PRX can be further modified at various sites (green, red, cyan) to impart desirable functionalities according to the application

Polyrotaxanes are molecular assemblies resembling a beaded-chain but at molecular scales [16, 17] (Fig. 1). The chain component is usually made from long chain polymers such as poly(ethylene glycol) (PEG) and poly(propylene glycol) (PPG). This acts as the axle component for the molecular assembly on which the ring molecules can exhibit various motions. Some of the ring or bead molecules used to synthesize PRXs are cyclodextrins (CD), crown ethers, or metal coordination complexes [16, 18]. However, the most commonly used PRXs for biological application are based on PEG–CD supramolecular assemblies, as both of these components are well-known biocompatible and biodegradable materials. The CDs are threaded onto the PEG chain in aqueous environment after which bulky end groups are added to prevent the coming out of CDs. The complexation of CDs with PEG is governed by van der Waals forces between CDs and PEG as well as intermolecular hydrogen bonds of CDs. The mobility of CDs along the PEG chain is expected after diminishing the hydrogen bonds by chemical modifications such as methylation. The CD rings can shuttle along the PEG chain based upon hydration or other stimuli. The extent of shuttling of the CD rings can be controlled by the number of CD per PEG chain, thereby controlling the molecular mobility. In such architecture, the molecular mobility is inversely correlated with the number of CD/PEG chain, i.e., lesser the number of CDs per PEG, the CD rings can move extensively resulting in high molecular mobility. However, when more numbers of CDs are threaded onto a PEG chain, the space for the movement of CDs is restricted leading to low molecular mobility [19]. This molecular mobility or the mobility factor has been quantified using methods such as wet and dry contact angle measurements and quartz crystal microbalance dissipation (QCM-D) studies [20, 21]. Furthermore, some researchers have shown that these CD rings can also be crosslinked to act like pulleys for the polymeric chains, which leads to the formation of very highly stretchable biomaterials [22]. Further, the CDs or the axle polymeric chains can accept numerous chemical modifications and conjugation of peptides, vitamins, or carbohydrates, which paves the way for the wide field of applications [23–26]. This brief review will cover the PRXs made from the CD-PEG complexes as these are the most widely explored towards biomaterial applications.

## Versatility of PRXs

PRXs are complex supramolecular assemblies which allow us the ability to modify several parameters such as the choice of axle polymer, molecular weight of the axle polymer, the number of CDs per polymer chain, addition of side chains to the axle polymer, modification of the CD with functional groups, addition of side chains to the CDs, and/or conjugation of peptides to the CDs [27, 28]. The presence of multivalency and molecular mobility in PRX supramolecular assemblies is also one of the key mechanisms which leads to improved efficiency of cell-ligand interactions [23–25]. Some of the commonly used polymers for the axle include but not restricted to PEG, PPG, poly(dimethylsiloxane), poly(ε-lysine), poly(ε-caprolactone) (PCL), poly(L-lactic acid), and nylon. CDs can be composed of six, seven, or eight glycosyl units, and they are named as α-CD, β-CD, and γ-CD respectively. The choice of determining the suitable axle polymer and the type of CD usually depends on the cavity diameter of the CD. For example, PEG, PCL, and poly(adipate)-based PRXs can be formed by complexing with α- and γ-CD only; however, when PPG or poly(1,3-dioxolane) is chosen as the axle polymer, all three forms of CDs can be threaded [29].

PRXs can be processed into scaffolds with controllable pore sizes. This helps to widen the utility for tissue engineering applications. PEG hydrogel scaffolds were produced by crosslinking hydrolyzable PRXs and also with cholesterol functionalized PRXs which were explored for cartilage regenerative applications. Hydrolyzable PRX-scaffolds containing hydroxyapatite particles were also synthesized and were suggested to exhibit promising outcomes for bone tissue engineering. PRXs can be utilized as crosslinkers for the poly(*N*-isopropylacrylamide) (PIPAAm) polymeric networks, thereby yielding a thermoresponsive and extremely stretchable hydrogel, which could be used for muscle tissue engineering [22, 30]. Similar high elastic hydrogels can be prepared by crosslinking acrylate monomers with slide-ring PRXs by exploiting the pulley effect [31]. Self-healing hydrogels can also be synthesized by creating a reversible bond between the ring molecules of PRXs and poly (acrylamide) [32]. Various stimuli-responsive PRX hydrogels, such as pH-sensitive, thermo-sensitive, photo-sensitive, etc., have been synthesized which could be potentially used for the on-demand delivery of drugs, peptides, or genes. These are usually synthesized by adjusting the threading CDs and the length of the polymeric axle, making chemical substitutions in the PRXs or by introducing stimuli-sensitive groups, which either breaks down or changes the conformation upon the necessary stimuli [33, 34]. For example, self-assembled PRX particles with disulfide bonds that could degrade and release the encapsulated drugs in the reducing environment have been developed [35]. Azo-substituted PRXs with PIPAAm exhibited responses to heat and UV stimuli [36]. Improving upon this, by introducing both a photo-initiator molecule and a photolabile group into the PRX crosslinker-based resin, it was shown that, upon exposure to 450-nm light, the PRX-based resin hardened, but when the hardened resin was irradiated with 250–300 nm UV light, the tensile strength of the resin reduced significantly indicating the photolabile nature [37]. Apart from these, functional group modifications such as acetylation, methoxylation or sulfonation, and conjugation of PRXs with peptides such as RGD, BMP2, and FGF2 have also been carried out on PRXs in order to improve cellular interactions [38–40]. With such numerous options to modify, improve, and incorporate unique features, PRXs can be considered to be one of the very versatile biomaterials allowing us to cater a wide array of clinical requirements.

## PRXs in directing stem cell commitment

Controlling the commitment of stem cells has a lot of implications in the field of tissue engineering and regenerative medicine. Growth factors, chemical supplements, siRNAs, and a combination of these have been studied extensively for the purpose of directing stem cell commitment [41–43]. It has been well established that the fate of stem cell differentiation also depends on the mechanical cues from the microenvironment [44]. Taking insights from this fact, PRXs with different molecular mobilities have been synthesized and their potential to alter the fate of stem cells were explored. Seo et al. explored the possibility of directing the MSCs into osteoblastic and adipocytic lineages by seeding them onto PRXs with low or high molecular mobilities, respectively [45]. It was found that molecular mobility influences the cellular response through focal adhesion kinases, actin organization, and RhoA-ROCK mechanosignaling pathway. On a high mobile PRX surface, the RhoA-ROCK signaling was inhibited, thereby directing the MSCs to enter into adipogenic differentiation; however, when the RhoA-ROCK was activated on the low mobile PRX surface, the MSCs differentiated into osteoblasts. Thereby, the possibility of using thin coatings of PRXs with different molecular mobility on the materials-surface to alter the stem cell commitment was established.

Extending upon this work, the effect of PRX molecular mobility was studied in the context of inducing cardiomyocytes from mouse iPS cells (iPSCs). Culturing iPSCs on PRXs with high mobility lead to the higher expression of Rac1 and N-cadherin expression indicating the strong cell-cell interactions when compared to cells cultured on gelatin-coated surfaces. The number of beating colonies indicating the successful cardiomyogenic commitment was higher on the high mobile PRX surface. Furthermore, the cardiomyocyte genes such as α-MHC, TnT2, and HCN4 were highly expressed in iPSCs cultured on high mobile PRXs confirming that PRX surface with high mobility could be effectively utilized to induce cardiomyocyte commitment from iPSCs [46].

Recently, it was reported that molecular mobility of PRXs could alter the stemness and differentiation capability of bone marrow-derived MSCs even during a short culture span [47]. It was found that immobilization of bFGF to sulfonated PRXs (S-PRXs) helped to increase the yield of MSCs and the molecular mobility played a major role in retaining the stemness of these cells. The MSCs cultured over high mobile S-PRX surfaces exhibited poor actin organization, retention of a key mechanosignaling element of YAP in the cytoplasm, and higher levels of stemness marker gene expression such as *Nanog* and *Oct4*. Further, when these MSCs were collected and replated onto regular tissue culture polystyrene plates, only the cells grown on high mobile S-PRX surfaces with bFGF were able to differentiate into osteoblasts.

## PRXs in enhancing osteogenic differentiation

Biomaterials for bone tissue engineering and enhancing osteogenic differentiation are of great importance as bone is the most widely transplanted tissue next to blood transfusions [48]. Although autografts remain as the gold standard for bone tissue repair, bone graft substitutes and alternative solutions to enhance the regenerative capacity of bone tissues are persistently being sought upon, owing to the practical limitations of autografts [49]. The molecular mobility and the ability of PRXs to be modified with various functional groups and complex with growth factors have been explored for the purpose of enhancing bone regeneration. Bone morphogenetic protein 2 (BMP2) was complexed with S-PRXs and its efficacy in osteogenic differentiation was evaluated [50]. A polyelectrolytic complex of S-PRX and BMP2 was synthesized by combining negatively charged S-PRXs with positively charged BMP2. Sulfonation of CDs in the S-PRX helps to enhance the activity of BMP2, mimicking the function of heparin, proving to be advantageous than heparin to be used in a clinical scenario since S-PRXs did not exhibit the anticoagulant activity. It was found that S-PRX/BMP2 complex significantly increased the osteogenic activity of MC3T3-E1 cells when compared to free BMP2 and heparin-BMP2 complex. The enhancement of osteogenic activity was due to S-PRX/BMP2 complex enhancing the tolerability to noggin-induced deactivation and also prolonging the Smad signaling. Further, these complexes were shown to significantly improve the bone regeneration in vivo, when it was encapsulated within collagen sponges and implanted into the mouse calvarial defects [51]. Extending upon this, it was shown that it is possible to immobilize BMP-2 onto the previously dried surface coating of S-PRXs. This possibly allows PRX to be stored for a longer duration and immobilization of BMP2 can be done a few hours before the clinical application. The osteogenic differentiation of MC3T3-E1 cells was enhanced on the BMP2-immobilized S-PRX surface compared to free soluble form of BMP2 [52].

## PRXs for cartilage regeneration

Cartilage regenerative therapies mainly focus on finding suitable biomaterial scaffolds to support the growth of chondrocytes and aid the secretion of extracellular matrix proteins in a spatiotemporally controlled pattern for matching the native healthy tissue as close as possible [53]. Lee et al. synthesized a porous hydrogel which consists of PEG crosslinked with hydrolyzable PRX [54]. The scaffold showed promising results for the proliferation of the chondrocytes in vitro. Further, the microporous hydrogel scaffold was able to trap a higher number of chondrocytes due to the primary amine functionalization in the PRXs [55]. The erosion rate of the PRX-based hydrogel scaffold was also controllable, thereby providing ample time for the chondrocytes to produce of glycosaminoglycan (GAG), indicative of promising use in cartilage regeneration. A similar hydrogel scaffold was synthesized by introducing cholesterol moieties in the PRXs, which has the advantage to mimic the lipid layers in the cell membrane, thereby improving the biocompatibility [56]. It also helped to control the Young’s modulus and the rate of degradation of the PRX-scaffold. It was found that the GAG content increased with the addition of cholesterol groups up to 15% degree of substitution. With the ability to retain huge amounts of water, form interconnected porous structures, and tune the mechanical properties such as the Young’s modulus and the degradation rates, PRX-based scaffolds are interesting candidates for cartilage regeneration.

## PRXs for other tissue engineering applications

Cellular adhesion is one of the most important requirements for biomaterial interfaces in the tissue engineering approach. It has been found that the molecular mobility of PRXs could be used to alter the cellular adhesion patterns. Generally, PRXs with low mobility tend to enhance the cellular adhesion and spreading of adherent cells such as fibroblasts, MC3T3-E1, and MSCs [47]. This could be because the low mobile PRX could mimic the mechanics of the native matrices of adherent cells leading to higher cell-biomaterial interactions. It could also be reasoned that due to excessive sliding of CDs in the high mobile PRXs, the cells may not achieve enough tractions for effective spreading seen on soft hydrogels, thereby inhibiting the other functions such as cell migration or proliferation [57]. Apart from utilizing the low molecular mobility of PRXs to enhance cellular adhesion, RGD peptides have also been conjugated to the dynamic CDs in the PRXs to synthesize cell adhesive hydrogel scaffold [58]. Seo et al. observed that the interaction with integrin was quicker and the cells exhibited larger initial adhesion area compared to RGD tethered onto non dynamic polymer [38, 39].

Formation of microvascular networks in implanted biomaterials is one of the key events for a successful tissue regeneration, as these microvessels are necessary for the efficient transport of cells, nutrients, and removal of the wastes [59]. It was observed that the molecular mobility of PRXs could be used to modulate the formation of microvascular networks [60]. Human umbilical vein endothelial cells (HUVECs) were cultured on VEGF-immobilized S-PRX surfaces, and they respond to different molecular mobility was studied. It was noted that low molecular mobility and VEGF immobilization enhanced the proliferation of HUVECs. The expression of genes required for endothelial network formation and angiogenesis, such as rhoA, pdgf, ang-1, and pecam-1, was increased in HUVECs cultured on low molecular mobile S-PRX surfaces with immobilized VEGF. This surface was also found to increase the number of endothelial networks formed in 5 days of HUVEC culture.

The effect of molecular mobility of S-PRXs on hepatocyte functions has also been explored by using HepG2 cells [61]. Heparin-binding epidermal growth factor-like growth factor (HB-EGF) was immobilized onto the S-PRX surfaces. The high mobile S-PRX surface showed retention of YAP in the cytoplasm which is required for the optimal proliferation, survival, and maintenance of hepatic functions, compared to the nuclear YAP localization in the low mobile S-PRX surface. Consistently, the albumin secretion, one of the key hepatic functions, was significantly higher in the cells cultured on high mobile S-PRX with immobilized HB-EGF, indicating that these surfaces could be useful in hepatocyte cultures and liver tissue engineering applications.

The molecular mobility of polyrotaxane surfaces also affected collagen fibrillogenesis. Although the extent of molecular mobility was independent of the absorbing amount of collagen, higher mobility of polyrotaxane surfaces preferentially induced rearrangement of adsorbed collagen and caused collagen fibrillogenesis. When PRX-coated substrates were implanted subcutaneously in rats, it was observed that the recruitment of macrophages at the implant site was suppressed by polyrotaxane surfaces with higher mobility. These results suggest that the molecular mobility of polyrotaxane helps suppress inflammation and promote regeneration in vivo [62].

## Conclusions and future directions

As briefly reviewed above, we could see that PRXs are a novel class of supramolecular assemblies which exhibit mechanical movements at a molecular scale. It has also been shown that these mechanical signals from the molecular mobility of PRXs could be harnessed and utilized for modulating various mechanosignaling pathways and thereby driving specific gene expressions (Table 1). One of the peculiar advantages of PRXs is that the effects of molecular mobility holds true at various scales, such as hydrogels, scaffolds, and even in thin coatings. This allows us to utilize PRXs in various forms for different clinical scenarios. Furthermore, with the other advantages of being able to synthesize from biocompatible and biodegradable precursors and the ability to add various chemical groups, peptides, growth factors, and vitamins to impart cell-specific functions in the field of tissue engineering in the future, it should also be noted that different types of cell or tissues could exhibit different responses even to the same kind of PRXs. Therefore, it is necessary to design and choose the appropriate type of PRXs for a specific application. However, it could also be foreseen that researchers might develop multilayered or multiscale constructs in which different segments of the construct could exhibit different molecular mobility, thereby closely mimicking the complex layers and mechanics of the real tissues. However, we could see that most of the studies have shown the intended effects of PRXs only in the in vitro stage, except for a handful of studies in vivo. Exploring the in vivo effects could possibly bring the interesting potentials of PRXs to the clinical benches. Thus, PRX-based biomaterials hold promising directions in the field of mechanobiology mediated tissue engineering.

**Table 1:** List of a few of the different modifications of PRX, the various processability of PRXs, and the effect of molecular mobility on cellular functions and their possible applications

No.	Type of PRX	Usage form	Type of study	Notable finding^1^	Possible application	Reference
1	Methylated PRX	Thin coating	In vitro; hMSC	H.Mf: ↓ RhoA signaling; L.Mf:↑ RhoA signaling	Modulating stem cell differentiation into adipogenic or osteogenic	[45]
2	Methylated PRX	Thin coating	In vitro; iPSC	H.Mf: ↑ Rac1, ↑TnT2; L.Mf:↓ Rac1, ↓TnT2	Enhancing cardiomyocyte differentiation from iPSCs	[46]
3	bFGF immobilized S-PRX	Thin coating	In vitro; hMSC	H.Mf: ↑ Nanog, ↑Oct4, Cytoplasmic YAP; L.Mf:↓ Nanog, ↓Oct4, Nuclear YAP	Retention of hMSCs stemness during expansion phase	[47]
4	BMP2/S-PRX	Polyelectrolyte complex	In vitro; MC3T3-E1	More tolerant to noggin deactivation, prolonged Smad signaling	Enhancing osteogenic differentiation	[50]
5	BMP2/S-PRX	Polyelectrolyte complex coated onto collagen scaffold	In Vivo; mouse cranial defect	Rapid bone regeneration	Potential bone graft substitute	[51]
6	BMP2 tethered S-PRX	Thin coating	In vitro; MC3T3-E1	Tethering BMP2 to S-PRX enhances cell adhesion and osteogenic gene expression	Enhancing osteogenic differentiation	[52]
7	PEG crosslinked with aminated PRX	Porous scaffold	In vitro; primary rabbit chondrocytes	Interconnected pores in scaffold; fast water absorption and swelling of hydrogel; very good cell adhesion	Potential scaffold for cartilage regeneration	[54]
8	PEG crosslinked with aminated PRX and terminal ester linkages	Porous scaffold	In vitro; primary rabbit chondrocytes	Enhanced trapping of cells; tunable degradation of scaffold; enhanced GAG production	Potential scaffold for cartilage regeneration	[55]
9	Cholesterol-modified PRX	Porous scaffold	In vitro; primary rabbit chondrocytes	Enhanced cell adhesion; tunable degradation of scaffold; enhanced GAG production	Potential scaffold for cartilage regeneration	[56]
10	RGD-conjugated PRX	Injectable scaffold	In vitro; L929 mouse fibroblasts	Enhanced biocomaptibility and cell adhesion	Injectable hydrogel scaffold	[58]
11	RGD-conjugated PRX	Thin coating	In vitro; NIH3T3	Rapid integrin binding; more cellular spreading	Improving ligand-cell interaction in biomaterial surfaces	[38]
12	VEGF-immobilized S-PRX	Thin coating	In vitro; HUVEC	L.Mf: enhanced cell proliferation; increased RhoA, Pdgf, ang1; increased endothelial network	Enhancing angiogenesis	[60]
13	HBEGF-immobilized S-PRX	Thin coating	In vitro; HEPG2	H.Mf: cytoplasmic YAP retention; increased albumin secretion	Proliferating functional hepatic cells; liver tissue engineering	[61]
14	RGD-conjugated PRX	Thin coating	In vitro; PC12	Increased cellular adhesion	Modulating neuronal differentiation	[63]
15	RGD-conjugated PRX	Thin coating	In vitro; P19CL6	Early formation of beating cardiomyocytes	Modulating cardiomyocyte differentiation	[63]
16	Methylated PRX	Thin Coating	In vitro; C2C12	Enhanced myogenesis related genes	Muscle regeneration	[64]
17	PRX-crosslinked collagen	Corneal membrane	In vivo; rabbit corneal injury	Enhanced remodeling of corneal epithelium and stroma	Corneal regeneration	[65]
18	Methylated PRX	Thin Coating	In vivo, rat subcutaneous implantation	H.Mf: induced collagen fibrillogenesis; suppressed recruitment of the macrophage	Collagen fibrillogenesis and inflammation control	[62]

^1^*H.Mf*, high molecular mobility; *L.Mf*, low molecular mobility; ↑, increased; ↓, decreased

## Abbreviations

PRX: Polyrotaxane
S-PRX: Sulfonated polyrotaxane
PEG: Poly(ethylene glycol)
PPG: Poly(propylene glycol)
CD: Cyclodextrin
QCM-d: Quartz crystal microbalance dissipation
PCL: Poly(ε-caprolactone)
PIPAAm: Poly(*N*-isopropylacrylamide)
BMP2: Bone morphogenetic protein 2
FGF2: Fibroblast growth factor 2
siRNA: Small interfering ribonucleic acid
MSC: Mesenchymal stem cell
RhoA: Ras homolog family member A
ROCK: Rho-associated protein kinase
iPSC: Induced pluripotent stem cell
Rac1: Ras-related C3 botulinus toxin substrate 1
α-MHC: Myosin heavy chain, α isoform
TnT2: Troponin T
HCN4: Hyperpolarization activated cyclic nucleotide gated potassium channel 4
YAP: Yes-associated protein
Oct4: Octamer-binding transcription factor 4
GAG: Glycosaminoglycan
HUVECs: Human umbilical vein endothelial cells
VEGF: Vascular endothelial growth factor
pdgf: Platelet-derived growth factor
ang-1: Angiopoietin-1
pecam-1: Platelet endothelial cell adhesion molecule 1
HB-EGF: Heparin-binding epidermal growth factor like growth factor
